# Integrated Bioinformatics Analysis Identified ASNS and DDIT3 as the Therapeutic Target in Castrate-Resistant Prostate Cancer

**DOI:** 10.3390/ijms25052836

**Published:** 2024-02-29

**Authors:** Ae Ryang Jung, Sun Shin, Mee Young Kim, U-Syn Ha, Sung-Hoo Hong, Ji Youl Lee, Sae Woong Kim, Yeun-Jun Chung, Yong Hyun Park

**Affiliations:** 1 Department of Urology, Seoul St. Mary’s Hospital, College of Medicine, The Catholic University of Korea, Seoul 06591, Republic of Korea; exactly@nate.com (A.R.J.); iris1771@hanmail.net (M.Y.K.); ushamd@catholic.ac.kr (U.-S.H.); toomey@catholic.ac.kr (S.-H.H.); uroljy@catholic.ac.kr (J.Y.L.); ksw1227@catholic.ac.kr (S.W.K.); 2 Department of Integrated Research Center for Genome Polymorphism, The Catholic University of Korea, Seoul 06591, Republic of Korea; sunshin@catholic.ac.kr (S.S.); yejun@catholic.ac.kr (Y.-J.C.); 3 Department of Microbiology, The Catholic University of Korea, Seoul 06591, Republic of Korea

**Keywords:** prostate cancer, castration-resistant prostate cancer, bioinformatics, RNA-sequencing, novel key genes, gene expression

## Abstract

Many studies have demonstrated the mechanisms of progression to castration-resistant prostate cancer (CRPC) and novel strategies for its treatment. Despite these advances, the molecular mechanisms underlying the progression to CRPC remain unclear, and currently, no effective treatments for CRPC are available. Here, we characterized the key genes involved in CRPC progression to gain insight into potential therapeutic targets. Bicalutamide-resistant prostate cancer cells derived from LNCaP were generated and named Bical R. RNA sequencing was used to identify differentially expressed genes (DEGs) between LNCaP and Bical R. In total, 631 DEGs (302 upregulated genes and 329 downregulated genes) were identified. The Cytohubba plug-in in Cytoscape was used to identify seven hub genes (*ASNS*, *AGT*, *ATF3*, *ATF4*, *DDIT3*, *EFNA5*, and *VEGFA*) associated with CRPC progression. Among these hub genes, *ASNS* and *DDIT3* were markedly upregulated in CRPC cell lines and CRPC patient samples. The patients with high expression of *ASNS* and *DDIT3* showed worse disease-free survival in patients with The Cancer Genome Atlas (TCGA)-prostate adenocarcinoma (PRAD) datasets. Our study revealed a potential association between *ASNS* and *DDIT3* and the progression to CRPC. These results may contribute to the development of potential therapeutic targets and mechanisms underlying CRPC progression, aiming to improve clinical efficacy in CRPC treatment.

## 1. Introduction

Prostate cancer (PCa) is one of the most common malignancies in men, and its incidence has been continuously increasing over the last few decades [1]. Common treatment options for patients with PCa include active surveillance, radical prostatectomy, radiation therapy, and androgen deprivation therapy (ADT). ADT is the standard treatment option for advanced and metastatic PCa [2]. Although bicalutamide is one of the most effective initial treatments for advanced PCa patients, almost all patients ultimately progress to the CRPC stage [2].

The median survival of CRPC patients is in the range of 15–36 months, and patients with CRPC have a poor prognosis [3]. For the past decade, taxane-based chemotherapy has been the treatment of choice for patients with CRPC [4]. Recently, novel androgen receptor (AR)-axis target agents such as abiraterone acetate, enzalutamide, apalutamide, and darolutamide have been developed and approved by the U.S. Food and Drug Administration. The development of second-generation antiandrogen treatment has significantly extended the overall survival of patients [5]. Despite these advances, the response to second-generation antiandrogen treatment remains temporary. Resistance to second-generation antiandrogen treatments develops quickly and results in clinical progression of the disease [6].

In the recent study, the mechanisms of progression to CRPC have been demonstrated, including AR dependence, PI3K-Akt-mTOR, glucocorticoid, and FOXO signaling [7,8]. Despite understanding these mechanisms, the research and clinical paradigms for treatment of PCa, including CRPC, remain focused on the blockade of the AR-axis signaling, and it is not sufficient to develop CRPC therapeutic strategies [9,10,11]. Thus, investigation regarding the mechanism of progression to CRPC and identification of its novel targets are urgently needed to improve treatment strategies for CRPC [6,12].

The discovery of a druggable therapeutic target is the most important step in establishing innovative treatment strategies. Despite the long timeframe and financial investments in this process, the success rate in clinical development has been low [13]. In the past decade, advancements in technologies such as next-generation sequencing (NGS)-based omics and bioinformatics approaches for NGS data analysis have allowed for the detailed characterization of individual features in tumors and cancer-related genes. These advancements offer valuable insight into the molecular mechanism underlying cancer occurrence, progression, and metastasis [14]. A better understanding of these mechanisms provides a good opportunity for discovering therapeutic targets in drug development [14,15]. The identification of potential genes related to CRPC development has been demonstrated [16,17]; however, whether they are sufficiently understood remains to be determined.

In this study, we performed mRNA sequencing (mRNA-seq) to investigate DEGs between LNCaP and Bical R. We explored bioinformatics analysis using their data to screen for crucial pathways and hub genes associated with the progression to CRPC and validated the expression of the hub genes in vitro. Furthermore, we also confirmed the prognostic usefulness or clinical significance of hub genes by correlating the validated hub genes in vitro with data from GSE32269 and GSE28403. In both the GSE32269 and GSE28403 datasets, the expression of *ASNS* and *DDIT3* was upregulated in CRPC samples rather than PCa. At the same time, relationships between disease-free survival (DFS) and validated hub gene expression were analyzed in PCa patients from The TCGA database, ensuring the stability and reliability of the results. The results of the present study will help elucidate the understanding of the molecular mechanism underlying progression to CRPC and serve as a potential therapeutic target for CRPC patients.

## 2. Results

### 2.1. Construction of Bical R Cells

**Figure 1:**
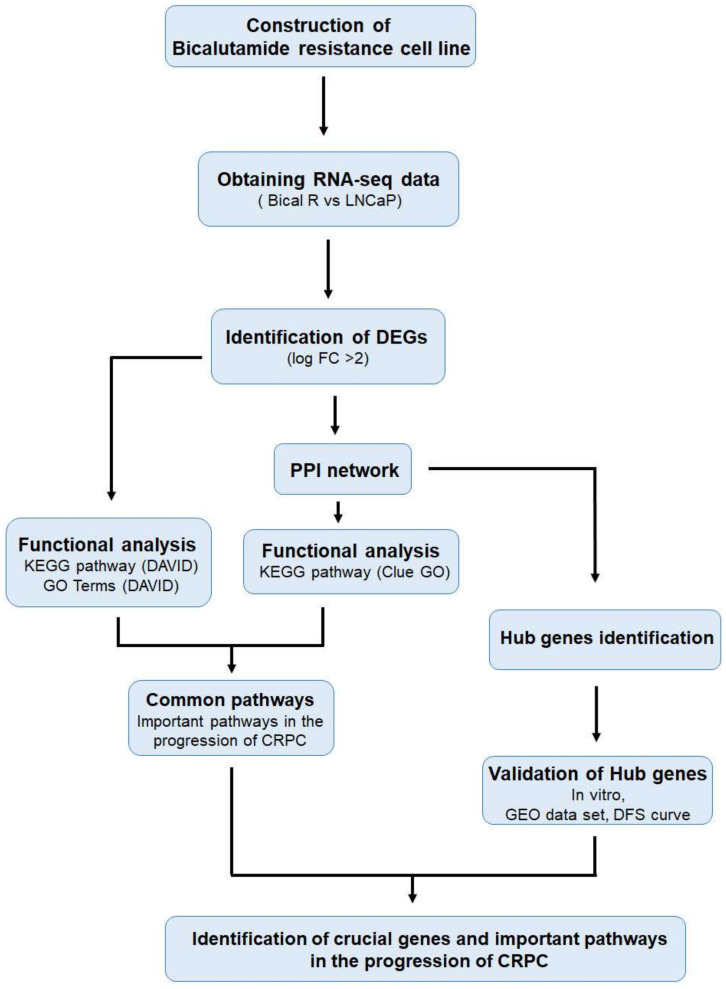
Schematic flow chart of current study for identification of crucial genes and important pathways in the progression of CRPC.

To investigate the crucial genes associated with the progression to CRPC, this study employed mRNA-seq, functional enrichment analysis, Protein-Protein interaction (PPI) analysis, identification of hub genes, and validation (Figure 1).

**Figure 2:**
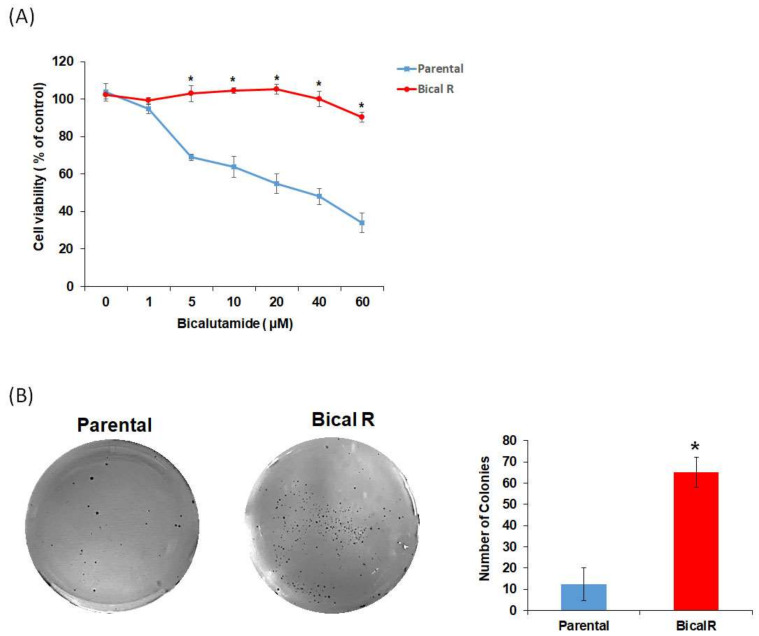
Construction of Bical R cell lines. Bical R cells derived from LNCaP were generated by treatment with bicalutamide (20 µM) over a period of 12 months. (**A**) Cell viability of parental cells (LNCaP) and Bical R cells was assessed using WST assay with indicated concentration of bicalutamide. (**B**) Cells were seeded and treated with bicalutamide for four weeks with the drug, and the regular media were replenished once every three days. The plates were stained with crystal violet, and the data are presented in viable colonies as mean  ±  SD. * indicates *p* < 0.05 compared with parental cells. All data are represented in triplicates. Statistical comparison between the two cell lines were analyzed using Student’s *t*-test.

To confirm that Bical R cells acquired resistance to bicalutamide compared with LNCaP parental cells, parental LNCaP and Bical R cells were treated with various concentrations of bicalutamide (1–60 µM), and cell viability was observed. The cell viability of LNCaP cells (IC_50_ = 16.18 µM) decreased in a dose-dependent manner, while there was almost no effect on cell viability of Bical R cells (IC_50_ = 42.40 µM) at the various concentrations (Figure 2A). Colony formation assays were also performed after treatment with bicalutamide (20 µM), revealing that the number of colonies in Bical R cells was significantly higher than that in parental LNCaP cells (Figure 2B). The results of both cell viability and colony formation assays indicated that Bical R cells were successfully constructed as bicalutamide-resistant cells. To determine whether bicalutamide resistance arises from loss of AR expression, we evaluated AR expression in parental LNCaP and Bical R cells. There was no statistical difference in AR expression between parental LNCaP and Bical R cells. These data show no alteration of AR expression upon acquisition of bicalutamide resistance, in which increased AR expression is not the sole determinant of progression to CRPC [18].

### 2.2. Functional Enrichment Analysis Based on DAVID

**Figure 3:**
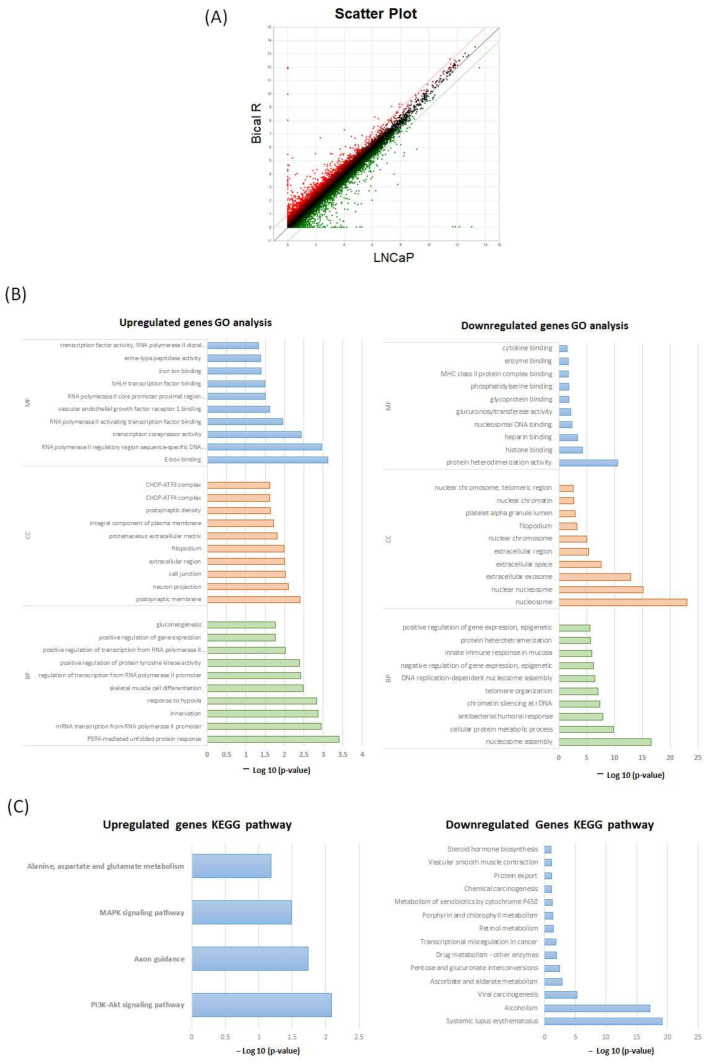
GO and KEGG pathway analyses for the significant DEGs. (**A**) Scatterplot shows the distribution of DEGs between Bical R and LNCaP. The X-Y axis represents log-2-transformed gene expression level. Each dot represents a gene, red dots are upregulated genes, and green dots are downregulated genes (|FC| > 2). Black dots do not represent expression differences. (**B**) The top 10 enriched GO of the upregulated and downregulated DEGs (BP—biological process; MF—molecular function; CC—cellular component). (**C**) KEGG pathway of upregulated and downregulated DEGs.

To identify DEGs between LNCaP and Bical R cells, we performed mRNA-seq and identified DEGs using the following cutoff value (|log FC| > 2.0). The scatter plot displays the distribution of DEGs by fold-changes (Figure 3A). A total of 24,424 DEGs were identified, including the 302 upregulated genes (red dots) and 329 downregulated genes (green dots) in the Bical R cells compared with the LNCaP cells. This is consistent with Western blotting; upregulated and downregulated genes did not include AR.

To extract more biological insights, we performed Gene ontology (GO) and Kyoto Encyclopedia of Genes and Genomes (KEGG) pathway analyses using The Database of Annotation Visualization and Integrated Discovery (DAVID). The enriched GO terms and KEGG pathways are shown in Figure 3B,C. In the enrichment analysis of molecular function (MF), upregulated genes were significantly enriched in the RNA transcription process, vascular endothelial growth factor binding, bHLH (basic helix-loop-helix) transcription factor binding, iron ion binding, and serine peptidase activity, while downregulated genes were significantly enriched in protein heterodimerization activity; protein-, lipid-, and DNA-binding processes; and glucuronosyltransferase activity. In the cellular compounds (CC) terms, upregulated genes were primarily associated with cellular anatomical entity terms and the CHOP (also known as DDIT3 and GADD153)–ATF4 complex and CHOP–ATF3 complex, while downregulated genes were linked to terms such as nucleosome, extracellular region, extracellular space, filopodium, platelet alpha granule lumen, nuclear chromatin, and nuclear chromosome. For biological processes (BP), the upregulated genes were PERK-mediated unfolded protein response, transcription DNA template, innervation, response to hypoxia, skeletal muscle cell differentiation, positive regulation of protein tyrosine kinase activity, and positive regulation of transcription and gene expression. The downregulated genes were nucleosome assembly, cellular protein metabolic process, antibacterial humoral response, chromatin silencing at rDNA, telomere organization, DNA replication-dependent nucleosome assembly, regulation of gene expression, and immune system processes.

According to KEGG pathway analysis, upregulated genes were significantly enriched in the PI3K-Akt, axon guidance, and MAPK signaling pathway. Downregulated genes were enriched in systemic lupus erythematosus, alcoholism, viral carcinogenesis, ascorbate and aldarate metabolism, pentose and glucuronate interconversions, drug metabolism, transcriptional misregulation in cancer, retinol metabolism, porphyrin and chlorophyll metabolism, and the metabolism of xenobiotics by cytochrome P450. Supplementary Tables S1–S3 illustrate the top 10 GO enrichment and KEGG pathways for the upregulated and downregulated genes.

### 2.3. KEGG Pathways Analysis of the Modules Based on ClueGO

We focused on the upregulated genes in Bical R cells compared with LNCaP cells to clarify the key genes implicated in the progression to CRPC. The PPI network of upregulated genes was constructed using the STRING online database (interaction score > 0.4) and visualized using Cytoscape version 3.8.0. In total, 210 nodes and 1422 edges were obtained. We applied the plug-in The Molecular Complex Detection (MCODE; a Cytoscape plug-in) to identify important modules in the PPI network, resulting in two clusters (cluster 1, MCODE score = 10.563; cluster 2, MCODE score = 5.795). Cluster 1 contained 102 genes, and cluster 2 contained 77 genes; both clusters may play a significant role in the progression to CRPC.

**Figure 4:**
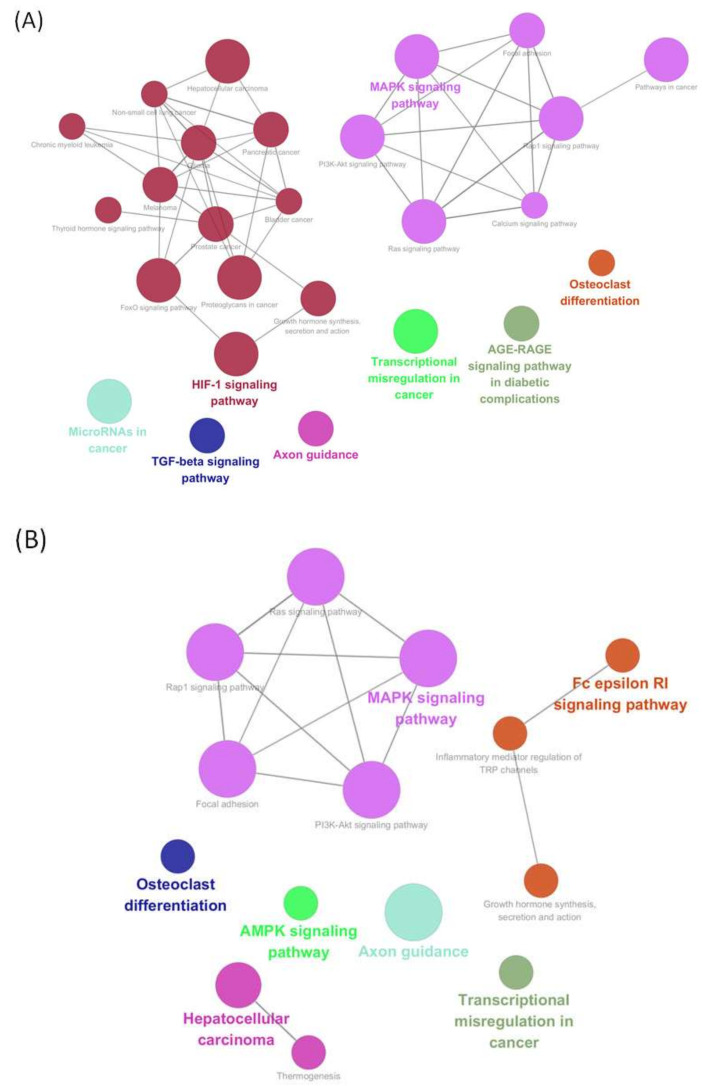
Identification of pathways based on ClueGO. We applied plug-in MCODE to identify important modules in the PPI network; the important pathway of each module (**A**) cluster1 and (**B**) cluster 2) is analyzed and visualized using the ClueGO including KEGG pathway. The enrichment shows only significant pathways (*p* < 0.05). The node size indicates *p*-value, and the node color code indicates the specific functional class in which they are involved. The bold fonts indicate the most critical functional pathways of each module.

We also conducted KEGG pathway analysis for each module using the Cytoscape plug-in ClueGO. Detailed information on the KEGG pathway analysis of each module is presented in Figure 4. Common significant pathways in modules 1 and 2 included the MAPK signaling, PI3k/Akt signaling, focal adhesion, Ras Signaling, Rap1 signaling, axon guidance, and transcriptional misregulation in cancer.

### 2.4. Identification of Hub Genes

The CytoHubba plug-in was employed to score and rank the nodes by degree, MNC, EPC, EcCentricity, and MCC methods [19] to identify 20 hub-forming genes (Supplementary Figure S1). The overlapping genes, determined through the five ranking methods in Cytohubba, were chosen as hub genes, including *ASNS*, *AGT*, *ATF3*, *ATF4*, *DDIT3*, *EFNA5*, and *VEGFA* (Table 1 and Supplementary Figure S1). Seven hub genes (*ASNS*, *AGT*, *ATF3*, *ATF4*, *DDIT3*, *EFNA5*, and *VEGFA*) were involved in cluster 1 (Supplementary Figure S2), and cluster 2 contained six hub genes (*ASNS*, *AGT*, *ATF3*, *ATF4*, *DDIT3*, and *EFNA5*).

**Figure 5:**
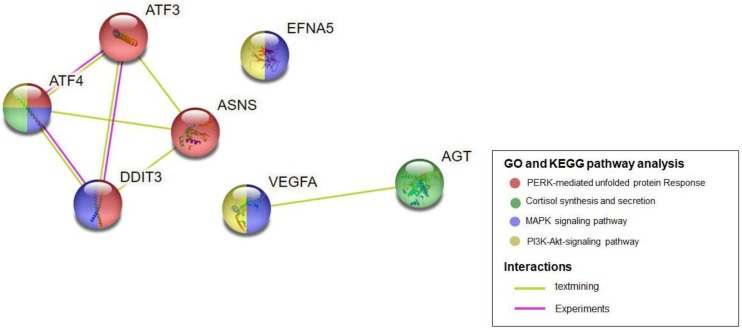
The hub genes identified in PPI network using the STRING online database.

PPI network analysis of hub genes was performed using STRING (Figure 5). The GO and KEGG pathway analysis of hub genes indicated that these genes were significantly enriched in the PERK-mediated unfolded protein response, cortisol synthesis and secretion, the MAPK signaling pathway, and the PI3K-Akt signaling pathway.

### 2.5. Validation of Hub Genes

**Figure 6:**
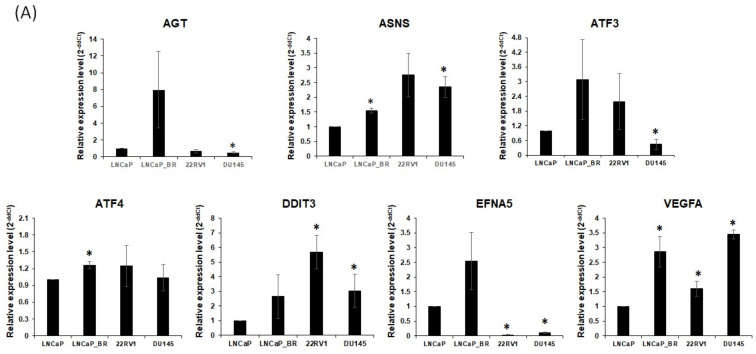

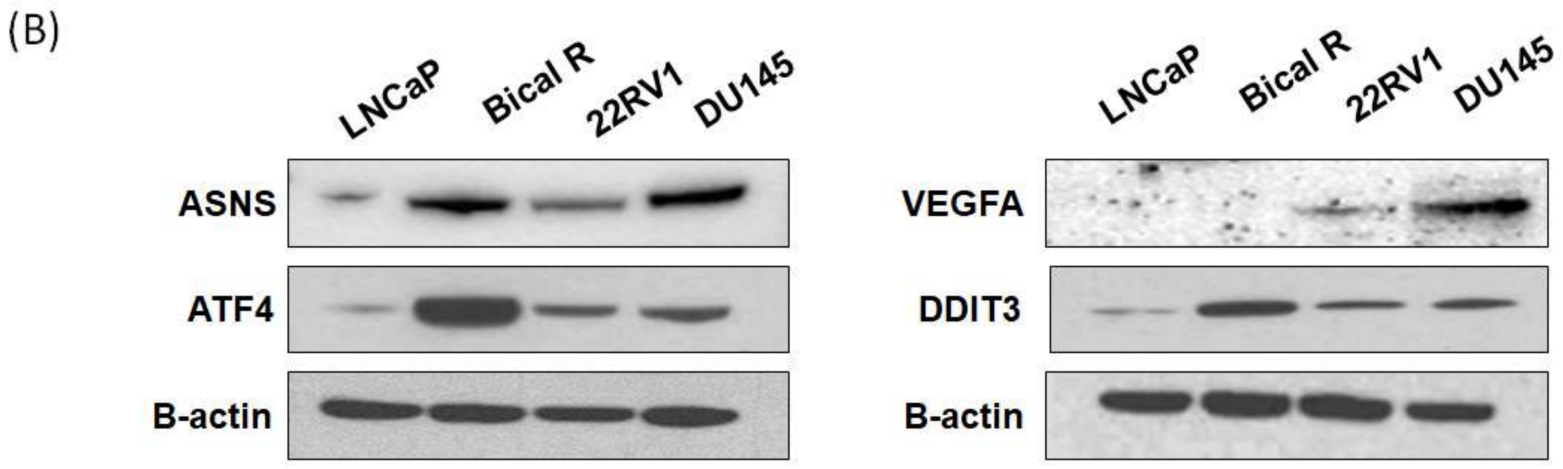
Experimental validation of hub genes. (**A**,**B**) The expression of hub genes in LNCaP, Bical R, 22RV1, and DU145 were confirmed using qRT-PCR and Western blot assay. * indicates *p* < 0.05 compared with LNCaP. All data are from four independent experiments with *n* = 3. *p*-values were determined by one-way analysis of variance (ANOVA).

To validate the expression of the hub genes associated with the progression to CRPC, we evaluated the expression of hub genes in LNCaP (a castration-sensitive PCa cell line), Bical R (a CRPC cell line from LNCaP), 22RV1 (a CRPC cell line with AR), and DU145 (a CRPC cell line with AR null). quantitative Real-Time PCR (qRT-PCR) analysis showed that the expression of *ASNS*, *ATF4*, and *DDIT3* was higher in Bical R, 22RV1, and DU145 than in LNCaP cells (Figure 6A). Western blotting analysis showed that the expression of *ASNS*, *ATF4*, and *DDIT3* was higher in Bical R, 22RV1, and DU145 than in LNCaP cells but not *VEGFA* (Figure 6B).

**Figure 7:**
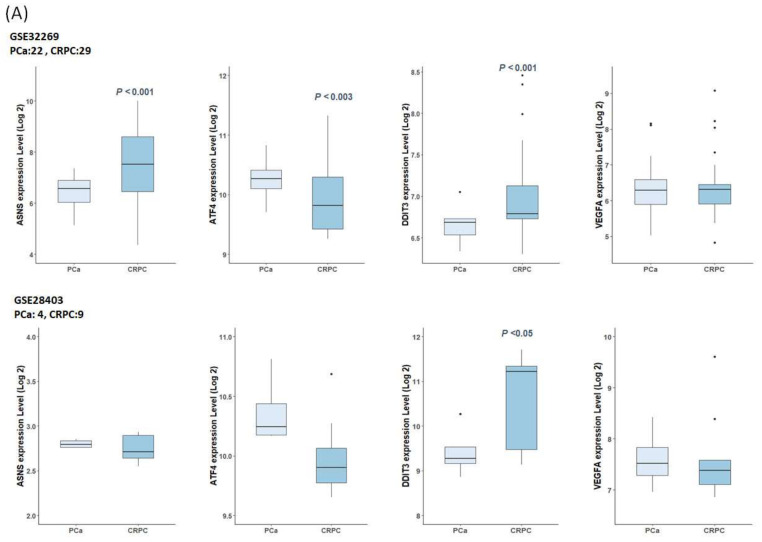

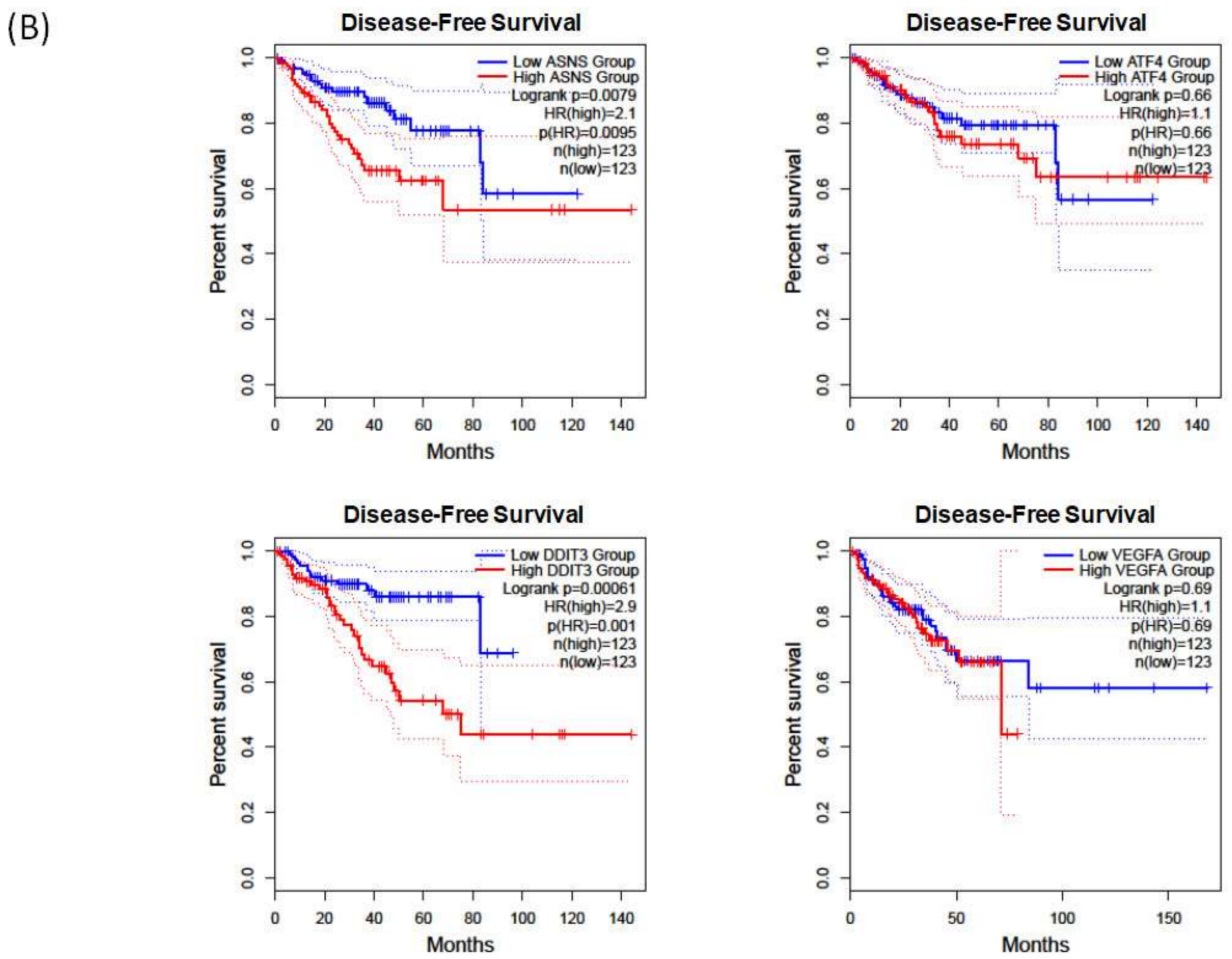
Expression levels of hub genes and prognostic values in public datasets. (**A**) Box plot showing expression levels of the *ASNS*, *ATF4*, *DDIT3*, and *VEGFA* between PCa and CRPC groups. GSE32269 datasets consisted of 21 localized primary PCa and 29 metastatic CRPC sample, and GSE28403 dataset consisted of four advanced PCa and nine CRPC samples, respectively. *p*-value was obtained by Welch’s two-sample *t*-test. (**B**) Relationship between the DFS of patients with PCa and hub genes expression in TCGA. The DFS curves with log-rank test were constructed by Kaplan–Meier method using Gene Expression Profiling Interactive Analysis (GEPIA2). Red line represents cases with high expression of the genes; blue line represents cases with low expression of the genes; dashed line represents the 95% confidence interval. Log-rank *p* < 0.05 was considered statistically significant. HR—hazard ratio.

Additionally, we verified the differential expression of four genes (*ASNS*, *ATF4*, *DDIT3*, and *VEGFA*) selected by in vitro experiments using the GSE32269 and GSE28403 datasets for external validation (Figure 7A). In the GSE32269 datasets consisting of 22 localized primary PCa and 29 metastatic CRPC samples, the expression of *ASNS* (*p* < 0.001) and *DDIT3* (*p* < 0.001) was upregulated in CRPC samples. In the GSE28403 dataset, the expression of *ASNS* and *DDIT3* was also upregulated in CRPC samples. However, the *DDIT3* expression differences observed in GSE28403 were only statistically significant. This may be attributed to the small number of patients (four PCa and nine CRPC samples), and some samples were collected from distant metastatic sites.

To elucidate the clinical usefulness of these four genes, we performed survival analysis. The disease-free survival (DFS) of patients with PCa from TCGA database was obtained according to the upper and lower quartiles of expression of the indicated genes. The results showed that high mRNA expression of *ASNS* (log-rank *p* = 0.0079) and *DDIT3* (log-rank *p* = 0.0006) was associated with poor DFS (Figure 7B). However, the expression of *ATF4* and *VEGFA* did not correlate with DFS.

## 3. Discussion

ADT, including bicalutamide, has been used to treat advanced PCa [20]. Most patients initially respond well to ADT, but a considerable number of patients experience relapse and resistance to ADT within a few years, which is known as CRPC [21]. Taxane-based chemotherapy (docetaxel), androgen-signaling-targeted inhibitors (abiraterone acetate and enzalutamide), and immunotherapy (sipuleucel-T) have been used for CRPC treatment. However, CRPC remains an incurable disease, for which approximately 15–25% CRPC of patients do not respond to abiraterone acetate or enzalutamide, and most patients treated with these agents develop acquired resistance during the clinical course [5]. Although the focus is still on the AR signaling pathway for the treatment of CRPC, there are limitations to understanding diverse etiological factors and molecular mechanisms in the progression to CRPC from early androgen-dependent PCa [11]. Therefore, it is necessary to identify important pathways and hub genes associated with the progression to CRPC, which might be a therapeutic target for CRPC.

In our study, there are intriguing aspects related to the mechanisms of progression to CRPC independent of the *AR*. First, we identified the following hub genes from the PPI network of upregulated DEGs as follows: *AGT*, *ASNS*, *ATF3*, *ATF4*, *DDIT3*, *EFNA5*, and *VEGFA*. Based on our validation results, *ASNS* and *DDIT3* exhibited higher expression in CRPC samples compared to PCa samples, and patients with higher expression of *ASNS* or *DDIT3* showed worse DFS. Therefore, we identified *ASNS* and *DDIT3* as genes playing a crucial role in the progression to CRPC. Also, these genes were involved in important modules and were related to PI3K-Akt signaling, MAPK signaling, and PERK-mediated unfolded protein response.

*ASNS* (asparagine synthetase), which catalyzes the synthesis of asparagine and glutamine from aspartate and glutamate in an ATP-dependent manner, is involved in the cell response to amino acid deprivation and other forms of cellular stress such as endoplasmic reticulum stress [22]. Recent studies have suggested that *ASNS* could have additional roles such as regulation of the mitotic spindle [23] and immunity [24], playing a diverse role in cancer progression [25,26]. Previous studies have demonstrated that *ASNS* is overexpressed in various cancers, including gastric, lung, and breast cancers. Consistent with findings, the overexpression of *ASNS* has been correlated with tumor growth, poor clinical outcome, chemoresistance, and metastasis [25,26,27]. In lung cancer cells and epidermoid carcinoma cells, *ASNS* knockdown reduces proliferation by arresting the Go/G1 cell cycle [28,29]. In breast cancer, *ASNS* is regulated by *IGF1/IGF2*, affecting amino acid transport, metabolism, protein biosynthesis, and stability [30]. Asparagine levels are directly correlated with *ASNS* expression regulating mTORC1 activity and protein synthesis. This indicates their crucial role in cell amino acid homeostasis, anabolic metabolism, and proliferation in cancer [31]. For example, in KRAS-mutant colorectal cancer cells, the high expression of *ASNS* induced by PI3K-AKT-mTOR pathways leads to metabolic shifts that sustain tumor growth [32]. Additionally, according to Sircar et al., the expression of *ASNS* was significantly increased in CRPC tissue compared to hormone-sensitive PCa- and normal prostate tissue. Knockdown of *ASNS* inhibited cell growth in both CRPC cell lines and the CRPC xenograft model [33]. H Wang et al. reported that inhibition of *ASNS* can lead to decreased viability in 22RV1 and DU145 cells [34]. This study hypothesized that *ASNS* overexpression may play an important role in the progression of advanced PCa to CRPC, which aligns with previous findings. Therefore, targeting *ASNS* could hold therapeutic potential for CRPC.

*DDIT3* (DNA damage-inducible transcript factor 3, also known as *CHOP* or *GADD153*) is a member of the CCAAT/enhancer binding protein (C/EBP) family and functions as a transcription factor that regulates gene expression, cellular growth, cellular differentiation, and energy metabolism. *DDIT3* plays an important role in the endoplasmic reticulum stress-induced apoptosis [35]. Notably, recent reports have shown that tumor-infiltrating CD8+ T cells show excessive *DDIT3* expression, which correlates with poor clinical outcomes in patients with ovarian cancer. Mechanistically, it has been demonstrated that upregulation of *DDIT3* blunts *tbx21* transcription in tumor-infiltrating CD8+ T cells, impairing T-cell effector immunity and indicating regulation of their antitumor activity [36]. Lin et al. reported that *DDIT3* expression is significantly increased in gastric cancer. In addition, our findings indicate a role for *DDIT3* in cancer stem cells. Overexpression of *DDIT3* promotes stemness of cancer stem cells in gastric cancer by regulating CEBPβ [37]. Zhang et al. showed that *DDIT3* also participates in PCa progression, including invasion capacity and cell proliferation. Regulation of *DDIT3* in PCa tissues may be a potential therapeutic target for PCa and CRPC [38]. These reports, together with our findings, suggest that *DDIT3* and *ASNS* may be core genes involved in the mechanism of progression to CRPC independent of the *AR*.

In our experiments, we found that *AR* is not differentially expressed, and the enriched GO terms and KEGG pathways are associated with non-AR signaling pathways. These results suggest that alternative activation pathways independent of the AR signaling are likely to be responsible for the progression to CRPC. Previous studies identified that CRPC shows a high incidence of somatic mutations, including PTEN-loss, p53/Rb loss, and Myc amplification/overexpression, which confers enhanced PI3K/Akt signaling and mTOR activation. It has been reported that the PI3K/Akt/mTOR pathway plays a pivotal role in the progression to CRPC from PCa and the maintenance of CRPC [17,39]. Mukherjee et al. reported that upregulation of the MAPK pathway is involved in the development of CRPC, as earlier studies have indicated a negative correlation between MAPK gene expression and time to biochemical relapse [40]. Recently, the activation of the MAPK/ERK signaling pathway was reported to promote enzalutamide resistance in PCa [41]. Our results are consistent with those of a previous study that showed the close association between the MAPK and PI3K/Akt signaling pathways and the development and maintenance of CRPC. These findings may provide insight into the mechanism for development of CRPC. 

However, there are still some limitations to this study. Firstly, only one cell line and its resistant variant were used for the identifying key genes associated with CRPC. This limitation is only partially alleviated by the validation results of hub gene candidates in LNCaP, Bical R, 22RV1, and DU145 cell lines. Additionally, further studies using tissue samples from CRPC are needed to validate the expression levels of hub genes candidates. However, owing to the difficulties in obtaining tissue samples from CRPC patients, we could not validate the results in clinical settings. To overcome these limitations, we analyzed and confirmed the expression levels of these genes using GSE datasets. We also confirmed the prognostic value of these genes using TCGA database. Although two key genes (*ASNS* and *DDIT3*) were identified as potential therapeutic targets and prognostic biomarkers for CRPC, further studies should investigate their role and the mechanisms in the progression to CRPC.

## 4. Materials and Methods

### 4.1. Cell Cultures

LNCaP and DU145 cells were purchased from the Korean Cell Line Bank (KCLB, Seoul, Republic of Korea). 22RV1 cells were purchased from American Type Culture Collection (ATCC; Rockville, MD, USA). LNCaP and 22RV1 cells were cultured in RPMI 1640 medium (Gibco; Thermo Fisher Scientific, Inc., Waltham, MA, USA) supplemented with 10% fetal bovine serum (FBS; Gibco) and Antibiotic-Antimycotic Solution (100×; Gibco) We generated Bical R cells in our laboratory by continuous exposure to bicalutamide (20 µM; Sigma-Aldrich, St. Louis, MO, USA) for 12 months. Bical R cells were routinely cultured with 10 µM bicalutamide to maintain resistance. DU145 cells were cultured in Dulbecco’s Modified Eagle Medium (Gibco) supplemented with 10% FBS, Antibiotic-Antimycotic Solution. All cells were incubated at 37 °C in a humidified atmosphere containing 5% CO_2_.

### 4.2. Drug Sensitivity Analysis by WST and Colony Formation Assays

In vitro drug sensitivity was determined using WST and colony-formation assays. The cells were seeded in a 96-well plate and incubated for 24 h. The cells were treated with different doses of bicalutamide (1–40 µM) for 48 h. Cell viability was measured using EZ-CYTOX (Daeil Lab Service Co., Ltd., Seoul, Republic of Korea) according to the manufacturer’s instructions.

For the colony-formation assay, cells were seeded into 6-well plates and treated with bicalutamide. The regular medium containing 20 µM of bicalutamide was replaced once every three days, and the cells were incubated for four weeks. The colonies were fixed and stained with 0.1% crystal violet. Viable colonies were counted.

### 4.3. mRNA Sequencing

Total RNA was extracted from each cells using TRIzol^®^ reagent (Invitrogen Life Technologies; Thermo Fisher Scientific, Inc., Waltham, MA, USA) following the manufacturer’s protocol. Total RNA was converted into libraries using a SMARTer Stranded RNA-Seq Kit (Clontech Laboratories, Inc., San Francisco, CA, USA). mRNA was isolated using a Poly(A) RNA Selection Kit (LEXOGEN, Inc., Vienna, Austria). The isolated mRNA was used for cDNA synthesis and shearing following the manufacture’ instructions. Indexing was performed using Illumina indexes 1–12. High-throughput sequencing was performed as paired-end 100 sequencing using HiSeq X10 (Illumina, Inc., San Diego, CA, USA).

### 4.4. Analysis of DEGs

Quality control of the raw sequencing data was conducted using FastQC; “http://www.bioinformatics.bbsrc.ac.uk/projects/fastqc/” (accessed on 30 October 2020). Adapter and low-quality reads (<Q20) were excluded using FASTX_Trimmer and BBMap. Then, trimmed reads were mapped to the reference genome using TopHat. Gene expression levels were estimated using fragments per kilobase per million reads (FPKM). The FPKM values were processed based on the quantile normalization method using EdgeR within R using Bioconductor [42]. Data mining and graphical visualization were performed using ExDEGA (E-biogen, Inc., Seoul, Republic of Korea). DEGs between LNCaP and Bical R samples were analyzed (|Log2FC| > 2.0).

### 4.5. GO and KEGG Pathway Analysis of DEGs

GO and KEGG pathway analyses of the selected DEGs were performed using the DAVID; “https://david.ncifcrf.gov”, accessed on 23 December 2020) [43]. GO comprised three categories: MF, BP, and CC. Statistical significance was set at *p* < 0.05.

### 4.6. Protein–Protein Interaction Network Analysis

STRING was performed to establish a PPI network of upregulated genes in Bical R cells, and validated interactions with confidence score > 0.4 were selected as significant. These genes were then imported into Cytoscape (version 3.8.0).

### 4.7. Screening of Module Genes and Kyoto Encyclopedia of Genes and Genomes Pathway Analysis of Upregulated DEGs

MCODE app was used to identify the most important module in the PPI network with a degree cut-off = 2, haircut on, fluff on, node score cutoff = 0.2, K-Core = 2, and max. depth = 100. Significant modules were identified with a MCODE score ≥ 4 and nodes ≥ 6. The ClueGO tool (a Cytoscape plug-in) was used to elucidate the KEGG pathways in different modules clustered with MCODE. KEGG pathway analysis was based on a cut-off value of <0.05.

### 4.8. Identification of Hub Genes

We sorted the genes in the entire PPI map according to the number of degrees. The CytoHubba application was used to identify hub genes in the PPI network as follows: the top 20 ranked by degree, MNC, EPC, EcCentricity, and MCC.

### 4.9. Quantitative Real-Time PCR

Total RNA was extracted suing TRIzol^®^ reagent (Invitrogen; Thermo Fisher Scientific, Inc., Waltham, MA, USA). Total RNA (1 µg) was reverse transcribed to cDNA using the PrimeScriptTM RT reagent Kit (Takara Bio Inc., Shiga, Japan), and qRT-PCR was performed using DreamTaq™ Green PCR Mast Mix (Thermo Scientific). The primer pairs used are listed in Supplementary Table S4. Relative gene expression levels were determined by normalization to GAPDH using the 2−ΔΔCT method.

### 4.10. Western Blot Analysis

Cells were lysed using RIPA buffer (Cell Signaling Technology, Danvers, MA, USA). Proteins were separated using Bolt Bis-Tris Plus gels (Thermo Scientific) and transferred onto a nitrocellulose membrane. The membranes were blocked for 1 h and then incubated with antibodies against ASNS (1:1000, Santa Cruz Biotechnology, Inc., Dallas, TX, USA), ATF4 (1:1000, Santa Cruz), DDIT3 (1:1000, Proteintech, Rosemont, IL, USA), VEGFA (1:500; proteintech), and β-actin (1:10,000, Santa Cruz). Membranes were incubated with horseradish peroxidase-conjugated horse anti-rabbit or anti-mouse IgG (GenDEPOT, Katy, TX, USA). Protein bands of protein were visualized using an ECL kit (DoGenBio, Seoul, Republic of Korea) and detected using an X-ray film.

### 4.11. Gene Expression in Public Datasets

The datasets were downloaded from the National Center for Biotechnology Information (NCBI) Gene Expression Omnibus (GEO) database (https://www.ncbi.nlm.nih.gov/geo/, accessed on 25 March 2021). The original gene expression profiles were obtained from the GSE32269 [44] and GSE28403 [45] datasets. GSE32269 consists of 22 localized PCa (hormone-sensitive) and 29 bone-metastatic CRPC, and GSE28403 consists of 4 advanced PCa (hormone-sensitive; samples were collected from distant metastatic site) and 9 CRPC (two samples were collected from distant metastatic sites). These datasets were generated using Affiymetrix Human Genome U133A and U133 Plus 2.0. The original expression data of these datasets were initially analyzed (background corrections, quartile normalization, and converted into gene expression measures) using R software version 4.0.4 with the Affy package and transferred log-2. The expression levels of each gene in PCa and CRPC tissues were visualized using R software with the ggplot2 package version 3.3.3.

### 4.12. Survival Analyses of TCGA-PRAD Datasets 

GEPIA2; “http://gepia2.cancer-pku.cn/” (accessed on 12 December 2021) is a web server for gene expression analysis, including differential genes, survival, and similar gene identification from the TCGA and GTEx databases [46]. GEPIA2 was used to evaluate the relationship between DFS and expression of each gene in TCGA-PRAD and patients with PCa grouped by upper and lower quartiles of expression of the indicated genes.

### 4.13. Statistical Analysis

The qRT-PCR data are expressed as mean ± standard deviation (SD) and were analyzed using IBM SPSS version 24.0 software (SPSS, Inc., Chicago, IL, USA). The data were subjected to the statistical analysis using one-way ANOVA. To compare the sample subgroups in gene expression of the GSE datasets, Welch’s *t*-test was performed for each gene. For DFS analysis and survival curves were plotted using the Kaplan–Meier method, and survival between two groups was assessed using the log-rank test, with 95% confidence interval added as dashed lines. Hazard ratios were calculated using the Cox proportional hazard model. Statistical significance was set at *p* < 0.05.

## Figures and Tables

**Table 1:** The top 20 hub genes in the CytoHubba plugin in Cytoscape (bold: overlapping genes).

Method									
Degree		MNC		EPC		EcCentricity		MCC	
Rank	Gene	Rank	Gene	Rank	Gene	Rank	Gene	Rank	Gene
1	***VEGFA***	1	***ATF4***	1	***VEGFA***	1	***DDIT3***	1	*GPC3*
2	***ATF4***	2	*GPC3*	2	***ATF4***	1	***ATF4***	2	*LTBP1*
3	***ATF3***	3	***EFNA5***	3	***DDIT3***	1	*CTH*	2	*IGFBP5*
3	*FYN*	3	***DDIT3***	4	***ATF3***	1	***ATF3***	4	*STC2*
3	***AGT***	3	***ATF3***	5	*FYN*	1	***ASNS***	5	*FAM20C*
3	*SREBF1*	3	***AGT***	6	***AGT***	1	*CYP2E1*	5	*RCN1*
7	***EFNA5***	7	*FYN*	7	*ENO2*	1	*ALDH2*	5	*MXRA8*
7	***DDIT3***	7	*LTBP1*	8	*MYOD1*	1	***VEGFA***	8	***ATF4***
7	*GPC3*	7	*IGFBP5*	9	***EFNA5***	1	*ENO2*	9	***ATF3***
10	*LTBP1*	10	***VEGFA***	10	***ASNS***	1	*PCK2*	10	***DDIT3***
10	*IGFBP5*	10	*FAM20C*	11	*DDIT4*	1	*GABBR1*	11	***VEGFA***
10	*GAD1*	10	*STC2*	12	*GPC3*	1	***AGT***	11	***AGT***
13	*ENO2*	10	*RCN1*	13	*GABBR1*	1	*SMARCD3*	13	*GABBR1*
13	*GABBR1*	10	*MXRA8*	14	*GAD1*	1	*MYOD1*	14	***EFNA5***
13	*MYOD1*	15	*EPHA3*	15	*IGFBP5*	1	*CHAC1*	14	***ASNS***
16	***ASNS***	15	***ASNS***	16	*LTBP1*	1	*DDIT4*	16	*ACKR3*
16	*RELN*	15	*SRGN*	17	*SREBF1*	17	*GABRA5*	16	*LPAR3*
16	*STC2*	15	*ACKR3*	18	*RELN*	17	*BEST1*	18	*CHAC1*
19	*CYP2E1*	15	*LPAR3*	19	*ACKR3*	17	***EFNA5***	18	*PCP2*
19	*FAM20C*	15	*GAD1*	20	*FAM20C*	17	*FSCN1*	20	*EPHA3*

## Data Availability

The original RNA-seq data are available from the corresponding author. Microarray data and RNA-seq data used in this study are already publicly available in GEO (https://www.ncbi.nlm.nih.gov/geo).

## Supplementary Materials

The following supporting information can be downloaded at: https://www.mdpi.com/article/10.3390/ijms25052836/s1.
