# Supplementary material for: Integrated Bioinformatics Analysis Identified ASNS and DDIT3 as the Therapeutic Target in Castrate-Resistant Prostate Cancer
Source: Int J Mol Sci. 2024 Feb 29;25(5):2836. doi: 10.3390/ijms25052836 (PMC10932076; doi:10.3390/ijms25052836)
Supplement: Supplementary file 1 [file ijms-25-02836-s001.zip › ijms-2854606-supplementary.pdf]

Supplementary Materials

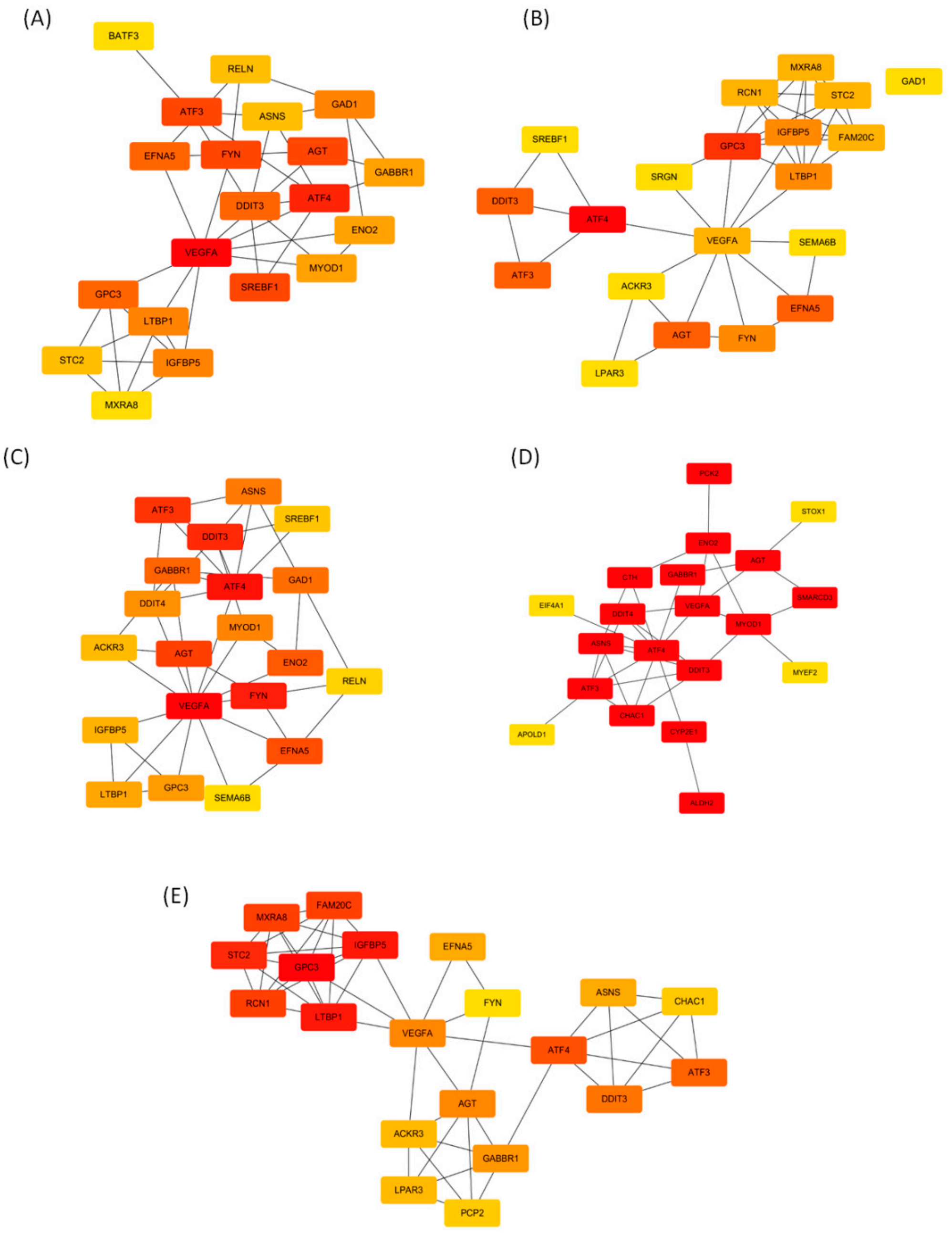

Supplementary Figure S1. The hub genes were screened out by five algorithms from CytoHubba. The protein-protein interaction network of hub genes shows the analysis results generated by the algorithms of (A) degree, (B) MNC, (C) EPC, (D) EcCentricity and (E) MCC. The red nodes represent high-scoring genes, wherease the yellow nodes represent low-scoring genes.

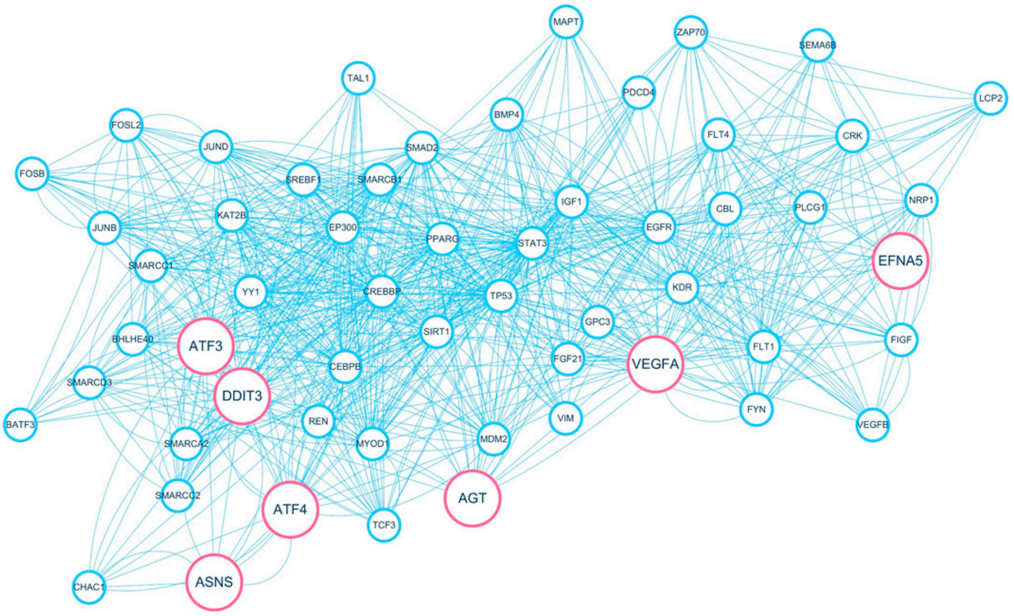

Supplementary Figure S2. The protein-protein interaction network of the upregulated genes analyzed using the STRING online database, identified hub genes and applied plug-in MCODE (MOCDE >3.0). Gene represented MCODE score >3.0. Cluster 1 involved all hub genes (AGT, ASNS, ATF3, ATF4, DDIT3, EFNA5 and VEGFA). Red representshub genes.

Supplementary Table S1. The top 10 GO terms of up-regulated genes

Category	GO_Term	*P*-Value	Associated Genes
BP	- PERK-mediated unfolded protein response	0.0004	ATF4, ATF3, ASNS, DDIT3
	- mRNA transcription from RNA polymerase II promoter	0.0012	SREBF1, ATF4, LMO2, DDIT3
	- Innervation	0.0014	SERPINE2, RNF165, GABRA5, POU4F1
	- Response to hypoxia	0.0015	VEGFB, ASCL2, PLOD2, VEGFA, APOLD1, WTIP, CD24, DDIT4, CITED2
	- Skeletal muscle cell differentiation	0.0032	MYOD1, HLF, ATF3, NUPR1, CITED2
	- Regulation of transcription from RNA polymerase II promoter	0.0038	SREBF1, MYOD1, HTATIP2, TCEAL2, ATF4, ATF3, NUPR1, SMARCD3, ZNF783, DBP, JUND, VEGFA, MAMLD1, POU4F1

	- Positive regulation of protein tyrosine kinase activity	0.0041	SRCIN1, AGT, RELN, CD24
	- Positive regulation of transcription from RNA polymerase II promoter in response to endoplasmic reticulum stress	0.0095	ATF4, ATF3, DDIT3
	- Positive regulation of gene expression	0.0173	SPRY2, STOX1, ATF4, VIM, VEGFA, POU4F1, PTGFR, FOXD1, CITED2
	- Gluconeogenesis	0.0175	ATF4, ATF3, ENO2, PCK2
MF	- E-box binding	0.0008	ASCL2, MYOD1, LMO2, BHLHE40, BHLHE41
	- RNA polymerase II regulatory region sequence-specific DNA binding	0.0011	MYOD1, HLF, STOX1, ATF4, ATF3, HMX1, LMO2, DBP, ZNF711, ALX4
	- Transcription corepressor activity	0.0036	BATF3, ATF3, CBX4, BHLHE40, WTIP, BASP1, BHLHE41, DDIT3, CITED2
	- RNA polymerase II activating transcription factor binding	0.0112	LMO2, BEX1, BHLHE40, BHLHE41
	- Vascular endothelial growth factor receptor 1 binding	0.0244	VEGFB, VEGFA
	- RNA polymerase II core promoter proximal region sequence-specific DNA binding	0.0312	ASCL2, BATF3, SREBF1, MYOD1, ATF4,<br>ATF3, JUND, BHLHE41, FOXD1, DDIT3
	- bHLH transcription factor binding	0.0320	LMO2, BHLHE40, BHLHE41
	- Iron ion binding	0.0402	PLOD2, HBQ1, CYP2E1, SCD5, HBE1, FTH1
	- Serine-type peptidase activity	0.0422	HTRA1, KLK4, RELN, PRSS16
	- Transcription factor activity, RNA polymerase II distal enhancer sequence-specific binding	0.0474	MYOD1, POU4F1, BHLHE40, BHLHE41
CC	- Postsynaptic membrane	0.0040	CNKSR2, SRCIN1, GRIN2C, NLGN4X, GABRA5,<br>GABBR1, NSG1, HTR3A, LIN7A
	- Neuron projection	0.0078	CNKSR2, ATF4, RAB39B, VIM, MAP2, POU4F1,SLC30A3, RGS17, LIN7A

	- Cell junction	0.0094	SRCIN1, GABRA5, PTPRR, GABBR1, BASP1, RGS17, KCNK1, TMEM163, GRIN2C, NLGN4X, SMAGP, SLC30A3, HTR3A
	- Extracellular region	0.0099	LTBP1, GABBR1, SERPINE2, VWDE, NPTX2, HTRA1, AGT, SPATA6, CRIM1, SRGN, STC2, RNASE4, KLK4, CA11, APOLD1, FGF21, PTGFR, COL5A1, EPHA3, VEGFB, MUCL1, CD163L1, FBLN2, LAMC3, C5ORF38, VEGFA, EGFL8, SUSD4, TMSB4X, EFNA5, IGFBP5
	- Filopodium	0.0104	SRCIN1, FSCN1, ABI2, IQGAP2, CD302
	proteinaceous extracellular matrix	0.0156	SMOC2, LTBP1, GPC3, FBLN2, LAMC3, GPC6, VEGFA, RELN, COL5A1
	- Integral component of plasma membrane	0.0189	IL27RA, GABBR1, TSPAN7, LPAR3, FXYD5, GPC3, BEST1, ROBO1, GRIN2C, GPC6, SLC18B1, SMAGP, SLC39A8, SLC30A3, SLC4A4, GABRA5, NPR3, PCDH7, KCNK1, PTGFR, ABCG1, EPHA3, SEMA6B, EPHA6, NLGN4X, SGCE, BMPR1B
	- Postsynaptic density	0.0235	CNKSR2, SRCIN1, FYN, GRIN2C, MAPT, NLGN4X, LIN7A
	- CHOP-ATF4 complex	0.0238	ATF4, DDIT3
	- CHOP-ATF3 complex	0.0238	ATF3, DDIT3

Supplementary Table S2. The top 10 GO terms of down-regulated genes

Category	GO_Term	*P*-Value	Associated Genes
**BP**	- Nucleosome assembly	3.05E-17	HIST4H4, HIST1H2BC, HIST1H2BD, HIST1H1C, HIST1H2BF, HIST1H2BG, NAP1L5, HIST2H3D, HIST2H2BC, HIST1H2BK, HIST1H2BL, HIST2H2BE, HIST2H2BF, HIST1H2BJ, HIST1H3A, HIST1H4E, HIST1H3D, HIST1H3E, HIST3H2BB, HIST1H4J, HIST1H3H, HIST1H4H
	- Cellular protein metabolic process	1.60E-10	ODAM, HIST4H4, KLK3, ADORA2A, IGF1, HIST2H3D, TTR, APP, SAA1, HIST1H3A, HIST1H4E, HIST1H3D, HIST1H3E, HIST1H4J, HIST1H3H, HIST1H4H

	- Antibacterial humoral response	1.09E-08	APP, HIST1H2BC, HIST1H2BD, ADM, HIST1H2BK, HIST1H2BF, HIST2H2BE, HIST1H2BG, HIST1H2BJ, SLPI
	- Chromatin silencing at rDNA	4.61E-08	HIST4H4, HIST1H3A, HIST1H4E, HIST1H3D, HIST1H3E, HIST1H4J, HIST1H3H, HIST1H4H, HIST2H3D
	- Telomere organization	8.46E-08	HIST4H4, HIST1H3A, HIST1H4E, HIST1H3D, HIST1H3E, HIST1H4J, HIST1H3H, HIST1H4H
	- DNA replication-dependent nucleosome assembly	3.01E-07	HIST4H4, HIST1H3A, HIST1H4E, HIST1H3D, HIST1H3E, HIST1H4J, HIST1H3H, HIST1H4H
	- Negative regulation of gene expression, epigenetic	5.45E-07	HIST4H4, HIST1H3A, HIST1H4E, HIST1H3D, HIST1H3E, HIST1H4J, HIST1H3H, HIST1H4H, HIST2H3D
	- Innate immune response in mucosa	1.21E-06	HIST1H2BC, HIST1H2BD, HIST1H2BK, HIST1H2BF, HIST2H2BE, HIST1H2BG, HIST1H2BJ
	- Protein heterotetramerization	2.14E-06	HIST4H4, HIST1H3A, HIST1H4E, HIST1H3D, HIST1H3E, HIST1H4J, HIST1H3H, HIST1H4H
	- Positive regulation of gene expression, epigenetic	2.95E-06	HIST4H4, HIST1H3A, HIST1H4E, HIST1H3D, HIST1H3E, HIST1H4J, HIST1H3H, HIST1H4H, HIST2H3D
**MF**	- Protein heterodimerization activity	2.61E-11	HIST1H2AC, HIST2H2AA4, HIST4H4, HIST1H2AE, SDCBP2, TTR, BAK1, HIST1H2BK, HIST1H2BL, HIST1H2BJ, ADRA2A, HIST1H4E, HIST3H2BB, HIST1H4J, HIST1H4H, HIST1H2BC, HIST1H2BD, HIST1H2BF, HIST1H2BG, H2AFJ, HIST2H3D, HIST2H2BE, HIST2H2BF, HIST1H3A, HIST1H2AI, HIST1H3D, HIST1H3E, HIST1H2AM, HIST1H2AL, HIST1H3H
	- Histone binding	6.38E-05	HIST4H4, TONSL, HIST1H3A, HIST1H4E, HIST1H3D, HIST1H3E, HIST1H4J, HIST1H3H, HIST1H4H, HIST2H3D
	- Heparin binding	4.94E-04	APP, NRP1, SAA1, MMP7, ADAMTS1, FSTL1, SERPIND1, THBS1, FN1, CXCL10

	- Nucleosomal DNA binding	0.004133	HIST1H3A, HIST1H3D, HIST1H3E, HIST1H3H, HIST2H3D
	- Glucuronosyltransferase activity	0.007985	UGT2B17, UGT2B11, UGT2B15, UGT2B28
	- Glycoprotein binding	0.013904	AZGP1, PIP, ACE2, HSPA5, THBS1
	- Phosphatidylserine binding	0.015621	SYT4, OSBPL8, SYTL2, THBS1
	- MHC class II protein complex binding	0.02135	ATP1B1, CD74, HLA-DRA
	- Enzyme binding	0.021971	DDC, APP, THRB, ADORA2A, HIST1H2AI, SLPI, HSPA5, HIST1H2AM, HIST1H2AL, SLC27A2, GBP1
	- Cytokine binding	0.029586	NRP1, CD74, GBP1
**CC**	- Nucleosome	1.10E-23	HIST1H2AC, HIST2H2AA4, HIST4H4, HIST1H2AE, HIST1H2BK, HIST1H2BL, HIST1H2BJ, HIST1H4E, HIST1H4J, HIST1H4H, HIST1H2BC, HIST1H2BD, HIST1H1C, HIST1H2BF, HIST1H2BG, H2AFJ, HIST2H3D, HIST2H2BE, HIST1H3A, HIST1H2AI, HIST1H3D, HIST1H3E, HIST1H2AM, HIST1H2AL, HIST1H3H
	- Nuclear nucleosome	8.21E-16	HIST1H2BC, HIST1H2BD, HIST1H2BF, HIST1H2BG, HIST2H2BC, HIST1H2BK, HIST1H2BL, HIST2H2BE, HIST2H2BF, HIST1H2BJ, HIST1H3A, HIST1H3D, HIST1H3E, HIST3H2BB, HIST1H3H

- Extracellular exosome	1.31E-13	SNCG, ATP1B1, HIST2H2AA4, HIST4H4, THRB, PGC, CRABP2, UCHL1, MMP7, ECHDC1, FSTL1, SDCBP2, ASAH1, ACTG2, TTR, AZGP1, APP, SAA2, HIST1H2BL, SAA1, PLA1A, DPP4, KCNMA1, TMEM205, DDC, ACTA2, PROSC, H2AFJ, PIGR, NAPRT, SMO, NPC1, HIST2H2BE, HIST2H2BF, PLA2G2A, SLPI, LAMC1, SLC27A2, LCP1, HLA-DRA, MVP, COASY, HIST1H2AC, CAB39L, HIST1H2AE, CLU, UGDH, BROX, MME, CD74, CBR1, FAM213A, HIST1H4E, PIP, TSTA3, HSPA5, FGL1, THBS1, HIST1H4J, HIST1H4H, FN1, HIST1H2BC, HIST1H2BD, S100P, KLK3, HIST1H2BF, HIST1H2BG, VTA1, FUCA2, PSMB8, ACPP, HIST2H3D, LCN2, CBLC, PRADC1, LAMA3, PI3, HIST1H3A, HBZ, ACE2, HIST1H2AI, HIST1H3D, FCGBP, HIST1H3E, SERPIND1, HIST1H2AM, HPGD, HIST1H2AL, HIST1H3H
- Extracellular space	2.84E-08	ODAM, NRP1, MSMB, PGC, CLU, MMP7, HFE, FSTL1, ASAH1, SPINK8, CXCL10, AZGP1, TTR, ACTG2, APP, HIST1H2BK, SAA2, SAA1, HIST1H2BJ, PIP, THBS1, ANGPT2, FN1, HIST1H2BC, HIST1H2BD, CES1, ACTA2, KLK3, HIST1H2BF, HIST1H2BG, IGF1, PIGR, FUCA2, ACPP, LCN2, GCG, F5, SEMA4G, ADM, HIST2H2BE, ACE2, PLA2G2A, SLPI, LAMC1, SERPIND1, AGR2, LCP1
- Extracellular region	4.47E-06	ODAM, HIST4H4, CLU, MMP7, IFI30, FSTL1, FGF13, JAG1, CXCL10, AZGP1, TTR, COL26A1, APP, SAA1, HIST1H4E, PIP, PLA1A, THBS1, HIST1H4J, ANGPT2, HIST1H4H, FN1, TMEFF2, DEFB132, KLK3, GNRH2, MUC20, IGF1, HIST2H3D, LCN2, GCG, PRADC1, NPC1, LAMA3, F5, ADM, HIST1H3A, VSTM2L, ACE2, PLA2G2A, PLA2G7, HIST1H3D, HIST1H3E, SERPIND1, LAMC1, HIST1H3H, GBP1

- Nuclear chromosome	1.00E-05	HIST4H4, HIST1H3A, HIST1H4E, HIST1H3D, HIST1H3E, HIST1H4J, HIST1H3H, HIST1H4H
- Filopodium	5.36E-04	FAM65B, ACTG2, DNALI1, ACTA2, FGF13, LCP1, ACPP
- Platelet alpha granule lumen	0.001125	APP, F5, CLU, IGF1, THBS1, FN1
- Nuclear chromatin	0.001904	POLR3G, HIST1H2AC, HIST2H2AA4, THRB, HIST1H2AE, HIST1H2AI, H2AFJ, HIST1H2AM, HIST1H2AL, NRIP1
- Nuclear chromosome, telomeric region	0.002704	HIST4H4, HIST1H3A, HIST1H4E, HIST1H3D, HIST1H3E, HIST1H4J, HIST1H3H, HIST1H4H

Supplementary Table S3. KEGG pathway enrichment of upregulated- and downregulated genes

	**KEGG Pathway**	***P*-Value**	**Associated Genes**
**Up-regulation**	- PI3K-Akt signaling pathway	0.0082	VEGFB, ATF4, LAMC3, VEGFA, LPAR3, RELN, EFNA5, FGF21, PCK2, COL5A1, DDIT4
	- Axon guidance	0.0183	SEMA6B, EPHA6, FYN, ROBO1, EFNA5, EPHA3
	- MAPK signaling pathway	0.0324	ATF4, MAPT, JUND, PTPRR, FGF21, GADD45B, DDIT3, PLA2G4D
**Down-regulation**	- Systemic lupus erythematosus	6.33E-20	HIST1H2AC, HIST2H2AA4, HIST4H4, HIST1H2AE, HIST1H2BK, HIST1H2BL, HIST1H2BJ, HIST1H4E, HIST3H2BB, HIST1H4J, HIST1H4H, HIST1H2BC, HIST1H2BD, HIST1H2BF, HIST1H2BG, H2AFJ, HIST2H3D, HIST2H2BE, HIST2H2BF, HIST1H3A, HIST1H2AI, HIST1H3D, HIST1H3E, HIST1H2AM, HIST1H2AL, HIST1H3H, HLA-DRA
	- Alcoholism	7.68E-18	HIST1H2AC, HIST2H2AA4, HIST4H4, ADORA2A, HIST1H2AE, HIST1H2BK, HIST1H2BL, HIST1H2BJ, HIST1H4E, HIST3H2BB, HIST1H4J, HIST1H4H, DDC, HIST1H2BC, HIST1H2BD, HIST1H2BF, HIST1H2BG, H2AFJ, HIST2H3D, HIST2H2BE,

		HIST2H2BF, HIST1H3A, HIST1H2AI, HIST1H3D, HIST1H3E, HIST1H2AM, HIST1H2AL, HIST1H3H
- Viral carcinogenesis	5.81E-06	HIST4H4, HIST1H2BC, HIST1H2BD, HIST1H2BF, STAT5A, HIST1H2BG, BAK1, HIST1H2BK, HIST1H2BL, HIST2H2BE, HIST2H2BF, HIST1H2BJ, HIST1H4E, HIST3H2BB, HIST1H4J, HIST1H4H
- Ascorbate and aldarate metabolism	0.001483	UGT2B17, UGT2B11, UGDH, UGT2B15, UGT2B28
- Pentose and glucuronate interconversions	0.003169	UGT2B17, UGT2B11, UGDH, UGT2B15, UGT2B28
- Drug metabolism - other enzymes	0.010485	UGT2B17, CES1, UGT2B11, UGT2B15, UGT2B28
- Transcriptional misregulation in cancer	0.012943	MAF, HIST1H3A, ETV1, IGF1, HIST1H3D, HIST1H3E, HPGD, HIST1H3H, HIST2H3D
- Retinol metabolism	0.031594	DHRS3, UGT2B17, UGT2B11, UGT2B15, UGT2B28
- Porphyrin and chlorophyll metabolism	0.043511	UGT2B17, UGT2B11, UGT2B15, UGT2B28
- Metabolism of xenobiotics by cytochrome P450	0.049686	CBR1, UGT2B17, UGT2B11, UGT2B15, UGT2B28

Supplementary Table S4. qPCR primer pairs.

Gene	Forward sequence (5′ to 3′)	Reverse sequence (5′ to 3′)
AGT	AATGACCGCATCAGGGTGG	GGTTCAGGGTCACCTCCAAG
ASNS	AAGCCGAGGAGGAGAGTGAG	TCTCATTTCTGGTGGCAGAGAC
ATF3	ATGTCCTCTGCGCTGGAATC	TTGTTTCGGCACTTTGCAGC
ATF4	ATGGGTTCTCCAGCGACAAG	AGGGCATCCAAGTCGAACTC
DDIT3	TTGCCTTTCTCCTTCGGGAC	AAGCAGGGTCAAGAGTGGTG
EFNA5	ATGGGAATGTAACCGGCCTC	TGGGATTGCAGAGGAGATGTAG
VEGFA	CTTCAAGCCATCCTGTGTGC	TATGTGCTGGCCTTGGTGAG
GAPDH	GCTACAGCAACAGGGTGGTG	GGTCTACATGGCAACTGTGAGG
